# Validity of a Smartphone App to Objectively Monitor Performance Outcomes in Degenerative Cervical Myelopathy: Preliminary Findings From a Longitudinal Observational Study

**DOI:** 10.2196/52832

**Published:** 2024-09-09

**Authors:** Alvaro Yanez Touzet, Tatiana Houhou, Zerina Rahic, Ilya Laufer, Konstantinos Margetis, Allan R Martin, Nicolas Dea, Zoher Ghogawala, Misha Kapushesky, Mark R N Kotter, Benjamin M Davies, MoveMed

**Affiliations:** 1 School of Medical Sciences Faculty of Biology, Medicine and Health University of Manchester Manchester United Kingdom; 2 MoveMed Ltd Cambridge United Kingdom; 3 Division of Science New York University Abu Dhabi Abu Dhabi United Arab Emirates; 4 NYU Langone Health New York, NY United States; 5 Icahn School of Medicine at Mount Sinai New York, NY United States; 6 Department of Neurological Surgery University of California, Davis Davis, CA United States; 7 Department of Surgery University of British Columbia Vancouver, BC Canada; 8 Lahey Hospital and Medical Center Burlington, MA United States; 9 Department of Clinical Neurosurgery University of Cambridge Cambridge United Kingdom; 10 Department of Clinical Neurosciences Ann McLaren Laboratory of Regenerative Medicine Cambridge United Kingdom; 11 Department of Clinical Neurosciences University of Cambridge Cambridge United Kingdom; 12 See Acknowledgements

**Keywords:** validation study, patient outcome assessment, smartphone, neurology, psychometrics, validity, validation, outcomes, degenerative, myelopathy, neuroscience, spine, monitor, monitoring, neuromuscular, muscular, mHealth, apps, measure, measures, measurements

## Abstract

**Background:**

Developing new clinical measures for degenerative cervical myelopathy (DCM) is an AO Spine RECODE-DCM research priority. Difficulties detecting DCM, and changes in DCM, cause diagnostic and treatment delays in clinical settings and heightened costs in clinical trials due to elevated recruitment targets. Digital outcome measures can tackle these challenges due to their ability to measure disease remotely, repeatedly, and more economically.

**Objective:**

The study aims to assess the validity of MoveMed, a battery of performance outcome measures performed using a smartphone app, in the measurement of DCM.

**Methods:**

A prospective observational study in decentralized secondary care was performed in England, United Kingdom. Validity and risk of bias were assessed using criteria from the COSMIN (Consensus-Based Standards for the Selection of Health Measurement Instruments) manual. Each MoveMed outcome was compared with 2 patient-reported comparators, with a priori hypotheses of convergence or divergence tested against consensus thresholds. The primary outcome was the correlation coefficient between the MoveMed outcome and the patient-reported comparators. The secondary outcome was the percentage of correlations that aligned with the a priori hypotheses. The comparators used were the patient-derived modified Japanese Orthopaedic Association score and the World Health Organization Quality of Life Brief Version questionnaire. Thresholds for convergence or divergence were set at ≥0.3 for convergence, <0.3 for divergence, and >0/<0 for directionality.

**Results:**

A total of 27 adults aged 60 (SD 11) years who live with DCM and possess an approved smartphone were included in a preliminary analysis. As expected, MoveMed tests of neuromuscular function correlated most with questionnaires of neuromuscular function (≥0.3) and least with questionnaires of quality of life (<0.3). Furthermore, directly related constructs correlated positively to each other (>0), while inversely related constructs correlated negatively (<0). Overall, 74% (67/90) and 47% (8/17) of correlations (unidimensional and multidimensional, respectively) were in accordance with hypotheses. No risk-of-bias factors from the COSMIN Risk of Bias checklist were recorded. Overall, this was equivalent to “very good” quality evidence of sufficient construct validity in DCM.

**Conclusions:**

MoveMed outcomes and patient-reported questionnaires converge and diverge in accordance with expectations. These findings support the validity of the MoveMed tests in an adult population living with DCM. Criteria from COSMIN provide “very good” quality evidence to support this.

## Introduction

Abnormal limb movement is a key phenotype of disease affecting the nervous and musculoskeletal systems. Loss of dexterity, for example, is a notable manifestation of conditions such as Parkinson disease, degenerative cervical myelopathy (DCM), peripheral neuropathy, and osteoarthritis [1,2]. The significance of this phenotype can be seen in the physician’s approach to examining the neuromuscular systems, the features used to distinguish or measure its disease, or the information sought to define its care and research. Collectively, diseases affecting the nervous and musculoskeletal systems are estimated to account for 1.1 to 4.9 million deaths and 165 to 357 million disability-adjusted life year (DALYs) worldwide and are the leading causes of global disability, reflecting their often chronic nature [3-5].

While abnormal movement is a key component of diagnosis, it is also a key component of longitudinal monitoring, as these diseases typically lack responsive serological or imaging biomarkers [6]. Such monitoring is key to adjusting or reviewing treatment strategies over time and defining the success or failure of research trials [7]. Today, monitoring relies on qualitative outcome measures: classifications based on a hierarchy of exemplar functions, such as questionnaires or item selection. While qualitative tools can be robust, valid, and even performed by the patient remotely, their limited granularity and intrinsic subjectivity mean they lack accurate and responsive discrimination of small but significant changes, particularly for fluctuating diseases [8]. For clinical care, this means clinically important change is seen late, often at the cost of increased disability [9]. For clinical research, the low statistical power of qualitative tools means far higher sample sizes are needed for trials to mitigate type 2 errors.

This is exemplified within DCM, a slow-motion spinal cord injury estimated to affect 1 in 50 adults [10-13]. Here, dexterity, gait, and balance are key measurement constructs [14]. Currently, the gold-standard outcome measure is the modified Japanese Orthopaedic Association (mJOA) score, but it is poorly responsive [6]. Further, score variation, driven partly by the disease and partly by reliability, is more than twice the minimal clinically important difference. In practice, this demands sample sizes greater than 300 patients for 1:1 comparison with at least 80% power [15,16]. Developing new approaches to functional measurement is a recognized research priority [14].

Advances in our ability to assess limb performance can thus greatly improve our understanding of the patient’s clinical picture, lead to better decision-making and outcomes, as well as accelerate knowledge discovery [17,18]. The sensors contained within smartphones offer the potential to achieve this. Smartphones are increasingly carried by all patient groups, with far greater penetrance and priority than other wearable devices such as smartwatches [19]. Current focus in portable technology with respect to health has largely been on “background monitoring,” but shortcomings remain, including accurate and responsive insights at the individual patient level, as well as between-device variation [20].

This study evaluates MoveMed, a smartphone app originally developed by researchers from the University of Cambridge to assess hand, arm, and leg function in real-time, in the user’s natural environment, and under standardized conditions. This approach is, therefore, different from background monitoring: it harnesses the accuracy of mobile sensors to measure movement but does so during prescribed activities or tasks, designed by health care professionals and patients to target critical markers of disease. It can therefore be considered a patient-performed, performance-based outcome (PerfO) or performance-based outcome measure (PerfOM). Since MoveMed is being developed in accordance with ISO 13485 (Software as a Medical Device), testing of measurement properties was timely given recent laboratory experience of technological readiness (TRL4). In terms of V3 stages for biometric monitoring technologies [21], the testing in this paper corresponds to clinical validation.

MoveMed was originally developed for DCM. Therefore, the focus of this report is on the validity of the MoveMed battery of PerfOMs in DCM. However, recognizing that the measurement constructs in this disease are shared across other neuromuscular diseases, its validity is currently being explored in other conditions. Formal methods and criteria from the US Food and Drug Administration and COSMIN (Consensus-Based Standards for the Selection of Health Measurement Instruments) guidelines were used to design a prospective and decentralized observational study. Validity and risk of bias were principally assessed via hypothesis testing of construct validity. Content validity will be formally evaluated separately but is briefly described in this work. This paper is the first of a series of clinimetric studies about the measurement properties of MoveMed battery of PerfOMs.

## Methods

### Participants

**Figure 1:**
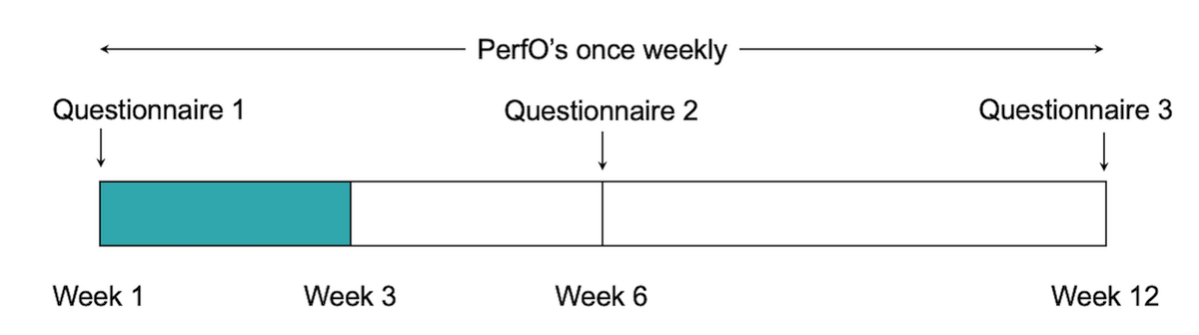
Study timeline. Data from the first 3 weeks of the study were included in this analysis due to the ongoing status of the trial. PerfO: performance-based outcome.

Between September 2022 and April 2023, a total of 27 people with DCM were enrolled in the prospective and decentralized EMPOWER study [22]. Prospective participants were recruited via a web-based campaign and asked to complete consent and registration forms (Figure 1) [23,24]. These were used to screen participants for eligibility. Participants were deemed eligible if they had a self-reported diagnosis of DCM, owned a smartphone, and were able to stand and walk without the assistance of another person. Eligible participants were invited to download the MoveMed app to their smartphones and complete an electronic, baseline questionnaire on neuromuscular function, hand dominance, and quality of life. This included questions from the patient-derived mJOA (P-mJOA) and the World Health Organization Quality of Life Brief Version (WHOQOL-Bref).

All enrolled participants were asked to perform each task in the MoveMed app once per week for a period of 12 weeks. Task adherence was remotely monitored once a week using a bespoke web-based dashboard. Participants were offered reminders and help via email once a week if 14 days passed since the completion of the latest task. These were offered a total of 2 consecutive times per participant, after which the participant was considered lost to follow-up. At weeks 6 and 12, participants were asked to complete the same electronic questionnaire from week 1.

### MoveMed and Tasks

**Figure 2:**
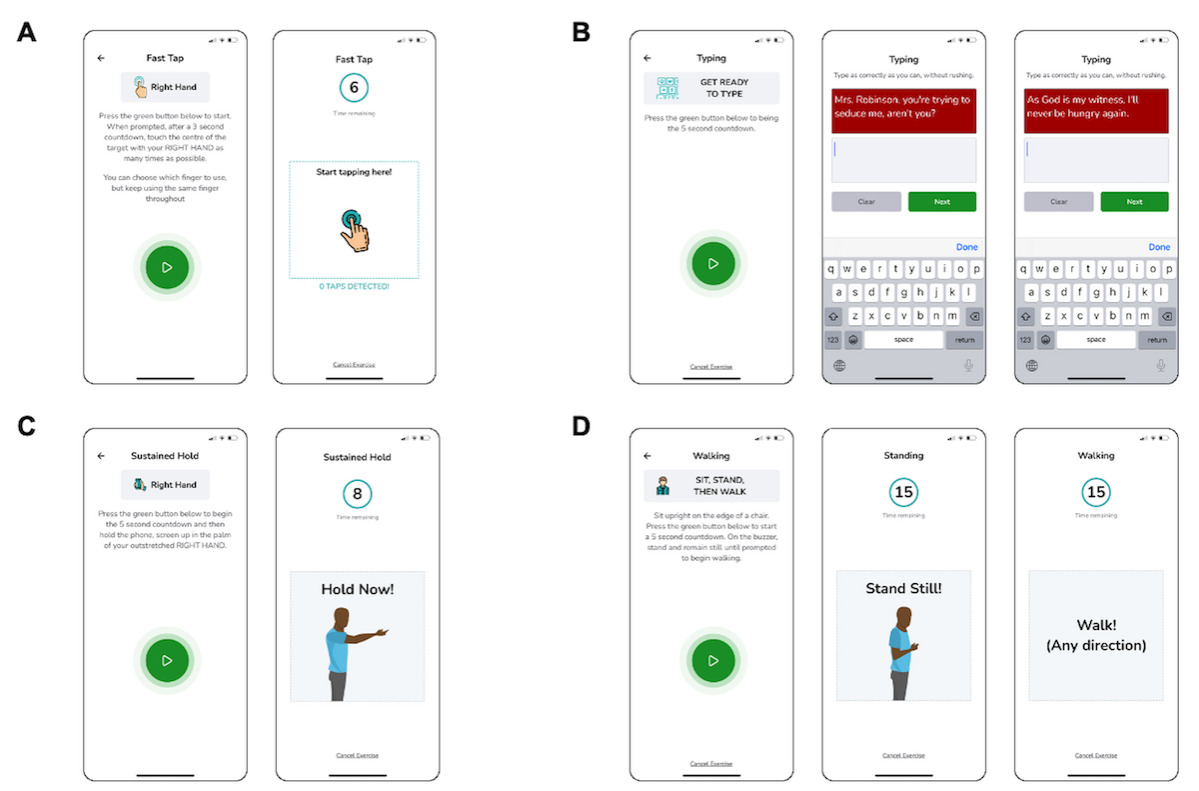
Schematic illustrations of the MoveMed battery of performance outcome measures. (A) The 6-second fast tap test; (B) the 2-stage typing test; (C) the 8-second hold test; and (D) the 15-second stand and walk test.

MoveMed is a smartphone app designed by academic neurosurgeons and computer scientists from the University of Cambridge to administer PerfOMs (Figure 2). These may be administered by clinicians during in-person visits or self-performed by individuals in the community. Version 1.0.0 of the app originally offered 3 performance tasks: a fast tap test, a hold test, and a stand and walk test. Version 1.2.2 incorporated an additional offering—a typing test—while making no changes to the 3 original tasks. Versions 1.0.0 and 1.2.2 were available in the Android Google Play Store and iOS App Store, respectively, at the time of writing and were used in this study by enrolled participants.

The fast tap test is a unidimensional PerfO task that assesses finger dexterity through a 6-second smartphone touch-based task. Users are shown a demonstrative cartoon (Figure 2A) and instructed to “touch the center of the target with [each] hand as many times as possible.” In-app video demonstration is also available. The construct (finger dexterity) is assessed by measuring the speed, accuracy, and efficiency of finger tapping as continuous variables and analyzing them as a panel of unidimensional measures. Content validity was assessed by AYT, MRNK, and BMD through literature review and clinical and patient input and deemed relevant, comprehensive, and comprehensible at the time of development [25-27]. In this study, tap latency was used as a reflective measure of finger dexterity.

The typing test is another unidimensional PerfO task that assesses finger dexterity through a 2-stage smartphone touch-based task. Users are shown a demonstrative cartoon (Figure 2B) and instructed to “type as correctly as they can, without rushing.” In-app video demonstration is also available. The construct (finger dexterity) is assessed by measuring the speed, accuracy, and efficiency of typing as continuous variables and analyzing them as a panel of unidimensional measures. Content validity was assessed by AYT, MRNK, and BMD through literature review and clinical and patient input and deemed relevant, comprehensive, and comprehensible at the time of development [25-27]. In this study, typing speed was used as a reflective measure of finger dexterity.

The hold test is a unidimensional PerfO task that assesses upper limb stability through an 8-second in-hand smartphone task. Users are shown a demonstrative cartoon (Figure 2C) and instructed to “hold the phone, screen up in the palm of [their] outstretched hand.” In-app video demonstration is also available. The construct (upper limb stability) is assessed by measuring the involuntariness, rhythmicity, and oscillation of the upper limbs as continuous variables and analyzing them as a multidimensional Stability Score. Content validity was assessed by AYT, MRNK, and BMD through literature review and clinical and patient input and deemed relevant, comprehensive, and comprehensible at the time of development [25-27]. In this study, the Stability Score was used as a reflective measure of upper limb stability.

The stand and walk test is a multidimensional PerfO task that assesses gait through a 2-stage in-hand smartphone task. During the first stage, users are instructed to “sit upright on the edge of a chair [and to] press the green button [when they are ready to] stand and remain still.” During the second stage, users are instructed to “walk [in] any direction.” In-app cartoons and video demonstrations are also available (Figure 2D). The construct (gait) may then be assessed by measuring standing or walking as continuous variables and analyzing them as multidimensional measures. Content validity was assessed by AYT, MRNK, and BMD through literature review and clinical and patient input and deemed relevant, comprehensive, and comprehensible at the time of development [25-27]. In this study, cadence was used as a reflective measure of gait.

### Patient-Reported Comparators

The 2 patient-reported outcomes (PROs) or PRO measures (PROMs) were used as comparators for DCM: the P-mJOA and the WHOQOL-Bref.

The P-mJOA score is a multidimensional, patient-reported questionnaire that assesses neuromuscular function in DCM across 4 items: motor dysfunction of the upper extremities (MDUE), motor dysfunction of the lower extremities (MDLE), sensory function of the upper extremities, and sphincter dysfunction [28]. Responses are scored on an ordinal scale per item and presented as both a panel of unidimensional scores and an unweighted sum-total, multidimensional score. The P-mJOA score was selected due to the existence of a systematic assessment of construct validity (*r*>0.5) and feasibility in DCM [6] and due to the use of its clinically reported analog (the mJOA) as the current gold standard. The P-mJOA score was favored over the mJOA score since it is intended to be a truly patient-reported equivalent of the mJOA score, which can be understood by individuals with no medical knowledge or training [29].

The WHOQOL-Bref is a multidimensional, patient-reported questionnaire that assesses quality of life across 26 items grouped into 4 domains: physical health, psychological health, social relationships, and environmental health [30]. Responses are scored on a 5-point ordinal scale per item and presented as a panel of sum-total, multidimensional scores. Responses to 2 items may, furthermore, be presented individually to give insight into the respondent’s global perception of their quality of life and their quality of health. These were presented in writing to describe the population’s characteristics but were not considered robust enough to warrant correlation analysis. The WHOQOL-Bref was selected due to the existence of systematic assessments of validity, reliability, and responsiveness in traumatic brain injury [31], Parkinson disease [32], and DCM [6]. It was also favored over the 36-Item Short Form Health Survey due to its relative brevity and over the EuroQOL Five Dimensions Questionnaire due to licensing restrictions.

### Statistical Analysis

The COSMIN manual defines validity as “the degree to which [an instrument] measures the construct it purports to measure” [33]. In the absence of a gold standard, validity may be assessed formally through hypotheses testing of correlations to known standards. These may then be judged both as a panel of stand-alone ratings [34].

In this study, we assessed validity by correlating the MoveMed PerfOs to their corresponding patient-reported comparators. This was achieved by comparing it to the P-mJOA and WHOQOL-Bref PROMs. Due to the ongoing status of the trial, data from the first 3 weeks of the study were included (Figure 1). All available tests within this period were included. Longitudinal replicates of MoveMed tasks were averaged before comparing their mean scores to the mean scores of the PROMs. Responses from the baseline questionnaire were used and results were subgrouped by diagnosis. Missing data were not imputed, and all analyses were done using Python (version 3.10.12; Python Software Foundation).

The goal of the analysis was to determine “whether the direction and magnitude of a correlation is similar to what could be expected based on the constructs that are being measured” [33,35]. Spearman rank correlation coefficients (*ρ*) were thus computed due to their suitability for ordinal scales. In accordance with COSMIN, *P* values were not used “because it is not relevant to examine whether correlations statistically differ from zero” [33,35]. Hypotheses about the direction and magnitude of correlations were instead drawn and adapted from COSMIN [33] and de Vet et al [36]. We hypothesized that the magnitude of correlations between outcomes measuring similar constructs should be ≥0.5; the magnitude of correlations between outcomes measuring related, but dissimilar, constructs should be ≥0.3, and ideally <0.5; the magnitude of correlations between outcomes measuring unrelated constructs should be <0.3; and the direction of correlations between outcomes measuring directly related constructs should be positive (>0) and negative (<0) between outcomes measuring inversely related constructs.

As reported in Yanez Touzet et al [6], constructs were defined as “similar” if they both measured the same domain with a unidimensional instrument. If they measured the same domain, but at least 1 of the instruments was multidimensional, the constructs were defined as “related but dissimilar.” Constructs measuring different domains were otherwise defined as “unrelated.”

### Risk-of-Bias Assessment

The COSMIN Risk of Bias checklist 9a [33] was used to assess the methodological quality of hypotheses testing.

### Overall Assessment

Overall assessments of construct validity were made using a panel of ratings and prior knowledge of content validity. These were appraised qualitatively and presented in writing due to the relatively higher importance of some comparators over others. As in COSMIN [33], correlations were converted into ratings by comparing results to hypotheses. Correlations in accordance with hypotheses were rated “sufficient.” Correlations in opposition were rated “insufficient.” Correlations in between boundaries (eg, *ρ*=0) and statistical artifacts (eg, nonmonotonic data) were rated “indeterminate.”

### Ethical Considerations

This study was independently assessed and approved by the University of Cambridge (HBREC.2022.13). All study participants provided informed consent before enrolling in the study and were able to opt out at any point. Study data were anonymized. None of the participants received any form of compensation for enrolling in or completing the trial.

## Results

### Participants

**Figure 3:**
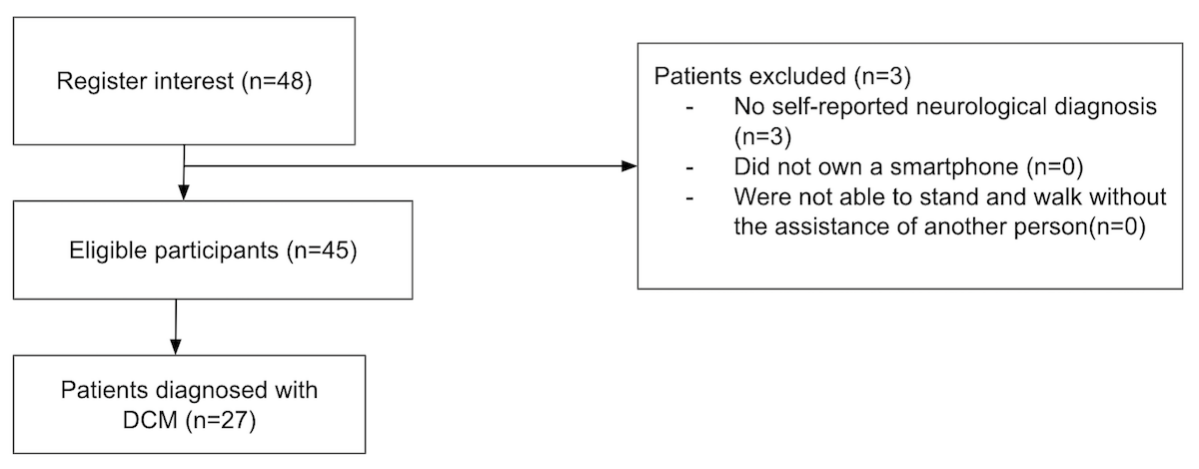
STROBE (Strengthening the Reporting of Observational Studies in Epidemiology) diagram. DCM: degenerative cervical myelopathy.

A total of 27 participants with DCM enrolled in the prospective and decentralized EMPOWER study (Figure 3), principally via advertisement through Myelopathy.org, a DCM charity [23,24]. On average, participants were aged 60 (SD 11) years (Table 1). DCM severity ranged from mild to severe (P-mJOA total score range 8-18). The impact on upper limb motor function ranged from none to “unable to eat with spoon but able to move hands” (P-mJOA MDUE subscore range 2-5) and the impact on lower limb motor function ranged from none to “able to move legs but unable to walk” (P-mJOA MDLE subscore range 2-7). Overall health perception ranged from “satisfied” to “very dissatisfied” (WHOQOL overall health range 1-4), and overall quality of life perception ranged from “very good” to “very poor” (WHOQOL overall quality of life range 1-5). In terms of the MoveMed PerfOs, participants paused for 80-2600 ms in between taps and typed approximately 0.6-2.5 keys per second. Arm stability ranged from 39% to 100% and cadence ranged from 14 to 112 steps per minute.

Differential app use was noted throughout the studied period (Table 2). More participants used the stand and walk and typing tests (n≥20) than the fast tap and hold tests (n≥12). However, mean adherence was higher with the fast tap and hold tests (100% and 90%, respectively) than with the stand and walk and typing tests (77% and 72%, respectively). Crucially, median adherence was satisfactory: 100% for the fast tap, hold, and stand and walk tests, and 80% for the typing test. Differential use was thus attributed to individual test preferences.

**Table 1:** Characteristics of study participants (N=27).

Feature		Value
Participants, n (%)		27 (100)
Age (years), mean (SD)		60.7 (10.8)
**P-mJOA^a^ score (reference range 0-18), mean (SD)**		11.4 (2.9)
	MDUE^b^ (reference range 0-5)	3.4 (1.1)
	MDLE^c^ (reference range 0-7)	4.1 (1.3)
	SDUE^d^ (reference range 0-3)	1.6 (0.8)
	SD^e^ (reference range 0-3)	2.3 (0.7)
**WHOQOL-Bref^f^ score, mean (SD)**		
	Overall QOL^g^ (reference range 1-5)	2.9 (1.1)
	Overall health (reference range 1-5)	2.6 (0.8)
	EH^h^ (reference range 8-40)	25.5 (5.5)
	PH^i^ (reference range 7-35)	18.6 (5.6)
	PS^j^ (reference range 6-30)	18.6 (4.1)
	SR^k^ (reference range 3-15)	9.2 (2.1)
MoveMed fast tap test intertap duration^l^ (s), mean (SD)		0.24 (0.13), 0.36 (0.52)
MoveMed hold test Stability Score^l^ (%), mean (SD)		78.6 (16.0), 75.8 (15.1)
MoveMed typing test speed (keys per second), mean (SD)		1.39 (0.42)
MoveMed stand and walk test cadence (steps per minute), mean (SD)		58.8 (26.9)

^a^P-mJOA: patient-derived modified Japanese Orthopaedic Association.

^b^MDUE: motor dysfunction of the upper extremity.

^c^MDLE: motor dysfunction of the lower extremity.

^d^SDUE: sensory dysfunction of the upper extremity.

^e^SD: sphincter dysfunction.

^f^WHOQOL-Bref: World Health Organization Quality of Life Brief Version

^g^QOL: quality of life.

^h^EH: environmental health,

^i^PH: physical health.

^j^PS: psychological health.

^k^SR: Social relationships.

^l^Data reported as “dominant hand, nondominant hand” mean (SD) pairs. Ranges are reported elsewhere in the paper.

**Table 2:** Correlations, ratings, and hypotheses for construct validity testing.

MoveMed outcome measure and comparator		Hypotheses			Result^a^	Total		Rating^b^			ROB^c^
		Direction	Magnitude				Direction		Magnitude		
**MoveMed fast tap test (rating proportion in correspondence: Direction 9/9, 8/9; Magnitude 4/9, 4/9)**											
	P-mJOA^d^ total score	*r* < 0	0.3 ≤ *|r*| (< 0.5)	–0.47, –0.42		12	+, +		+, +	No	
	P-mJOA MDUE^e^ subscore	*r* < 0	*|r*|≥ 0.5	–0.28, –0.40		12	+, +		–, –	No	
	P-mJOA MDLE^f^ subscore	*r* < 0	*|r*|< 0.3	–0.42, –0.18		12	+, +		–, +	No	
	P-mJOA SDUE^g^ subscore	*r* < 0	*|r*|< 0.3	–0.28, –0.36		12	+, +		+, –	No	
	P-mJOA SD^h^ subscore	*r* < 0	*|r*|< 0.3	–0.54, –0.44		12	+, +		–, –	No	
	WHOQOL^i^-Bref EH^j^ subscore	*r* < 0	*|r*|< 0.3	+0.21, –0.05		12	+, –		+, +	No	
	WHOQOL-Bref PH^k^ subscore	*r* < 0	*|r*|< 0.3	–0.39, –0.38		12	+, +		–, –	No	
	WHOQOL-Bref PS^l^ subscore	*r* < 0	*|r*|< 0.3	–0.64, –0.43		12	+, +		–, –	No	
	WHOQOL-Bref SR^m^ subscore	*r* < 0	*|r*|< 0.3	–0.15, –0.20		12	+, +		+, +	No	
**MoveMed hold test (rating proportion in correspondence: Direction 8/9, 9/9; Magnitude 5/9, 4/9)**											
	P-mJOA total score	*r* > 0	0.3 ≤ *|r*| (< 0.5)	+0.02, +0.55		13	+, +		–, +	No	
	P-mJOA MDUE subscore	*r* > 0	*|r*|≥ 0.5	+0.14, +0.64		13	+, +		–, +	No	
	P-mJOA MDLE subscore	*r* > 0	*|r*|< 0.3	+0.13, +0.36		13	+, +		+, –	No	
	P-mJOA SDUE subscore	*r* > 0	*|r*|< 0.3	+0.21, +0.47		13	+, +		+, –	No	
	P-mJOA SD subscore	*r* > 0	*|r*|< 0.3	–0.45, +0.14		13	+, +		–, +	No	
	WHOQOL-Bref EH subscore	*r* > 0	*|r*|< 0.3	+0.14, +0.31		21	+, +		+, –	No	
	WHOQOL-Bref PH subscore	*r* > 0	*|r*|< 0.3	+0.16, +0.43		21	+, +		+, –	No	
	WHOQOL-Bref PS subscore	*r* > 0	*|r*|< 0.3	–0.18, +0.02		21	–, +		+, +	No	
	WHOQOL-Bref SR subscore	*r* > 0	*|r*|< 0.3	+0.31, +0.47		21	+, +		–, –	No	
**MoveMed typing test (rating proportion in correspondence: Direction 9/9; Magnitude 7/9)**											
	P-mJOA total score	*r* > 0	0.3 ≤ *|r*| (< 0.5)	+0.38		20	+		+	No	
	P-mJOA MDUE subscore	*r* > 0	*|r*|≥ 0.5	+0.37		20	+		–	No	
	P-mJOA MDLE subscore	*r* > 0	*|r*|< 0.3	+0.21		20	+		+	No	
	P-mJOA SDUE subscore	*r* > 0	*|r*| < 0.3	+0.32		20	+		–	No	
	P-mJOA SD subscore	*r* > 0	*|r*| < 0.3	+0.07		20	+		+	No	
	WHOQOL-Bref EH subscore	*r* > 0	*|r*| < 0.3	+0.08		20	+		+	No	
	WHOQOL-Bref PH subscore	*r* > 0	*|r*| < 0.3	+0.17		20	+		+	No	
	WHOQOL-Bref PS subscore	*r* > 0	*|r*| < 0.3	+0.15		20	+		+	No	
	WHOQOL-Bref SR subscore	*r* > 0	*|r*| < 0.3	+0.10		20	+		+	No	
**MoveMed stand and walk test (rating proportion in correspondence: Direction 1/9; Magnitude 7/9)**											
	P-mJOA total score	*r* > 0	0.3 ≤ *|r*| (< 0.5)	–0.04		21	–		–	No	
	P-mJOA MDUE subscore	*r* > 0	*|r*|< 0.3	–0.17		21	–		+	No	
	P-mJOA MDLE subscore	*r* > 0	0.3 ≤ *r* (< 0.5)	+0.35		22	+		+	No	
	P-mJOA SDUE subscore	*r* > 0	*|r*|< 0.3	–0.18		21	–		+	No	
	P-mJOA SD subscore	*r* > 0	*|r*|< 0.3	–0.14		21	–		+	No	
	WHOQOL-Bref EH subscore	*r* > 0	*|r*|< 0.3	–0.28		21	–		+	No	
	WHOQOL-Bref PH subscore	*r* > 0	*|r*|< 0.3	–0.12		21	–		+	No	
	WHOQOL-Bref PS subscore	*r* > 0	*|r*|< 0.3	0.00		21	?		+	No	
	WHOQOL-Bref SR subscore	*r* > 0	*|r*|< 0.3	–0.31		21	–		–	No	

^a^Data reported as single “ρ” values or “dominant hand, nondominant hand” ρ pairs.

^b^“+”=Sufficient; “?”=Indeterminate. Data reported as single ratings or “dominant hand, nondominant hand” rating pairs; “–”=Insufficient.

^c^ROB: risk of bias.

^d^P-mJOA: patient-derived modified Japanese Orthopaedic Association.

^e^MDUE: motor dysfunction of the upper extremity.

^f^MDLE: motor dysfunction of the lower extremity.

^g^SDUE: sensory dysfunction of the upper extremity.

^h^SD: sphincter dysfunction.

^i^WHOQOL-Bref: World Health Organization Quality of Life Brief Version

^j^EH: environmental health.

^k^PH: physical health.

^l^PS: psychological health.

^m^SR: social relationships.

### Patient-Reported Comparators

Spearman rank correlation coefficients are reported in Table 2. As expected, correlations were positive between PerfOs and PROs measuring directly related constructs (eg, hold and typing tests vs P-mJOA and WHOQOL-Bref) and negative between PerfOs and PROs measuring inversely related constructs (eg, fast tap test vs P-mJOA and WHOQOL-Bref). This was most pronounced in the fast tap, hold, and typing tests.

Correlation magnitudes were, furthermore, highest between PerfOMs and PROMs of neuromuscular function (eg, fast tap test vs P-mJOA≥0.3) and lowest between PerfOMs and PROMs of quality of life (eg, fast tap test vs WHOQOL-Bref<0.3).

This was also in accordance with expectation and was most pronounced in the fast tap, hold, and typing tests.

Correlation magnitudes were notably low (<0.3) in the stand and walk test. This could be due to it being the only multidimensional PerfOM in the battery. Importantly, correlation with the lower limb comparator domain (ie, the P-mJOA MDLE subscore) was the highest, in accordance with expectation.

### Risk-of-Bias Assessment

No risk of bias factors from the COSMIN Risk of Bias checklist were recorded (Multimedia Appendix 1). This was equivalent to a “very good” rating for methodological quality [33].

### Overall Assessment

Hypotheses and result ratings are also reported in Table 2. These are appraised in writing due to the relatively higher weight of some comparators over others.

Overall, 74% (67/90) of correlations for the fast tap, hold, and typing tests were in correspondence with hypotheses (Table 2). This provides robust evidence for the validity of these PerfOMs in the assessment of DCM: particularly due to the relatively higher importance of the correlations to the upper limb comparator (ie, the P-mJOA MDUE subscore), which were concordant. For the stand and walk test, 47% (8/17) of the correlations were in correspondence with the hypotheses. This also provides preliminary evidence for the validity of this PerfOM in the assessment of DCM: particularly due to the relatively higher importance of the correlation to the lower limb comparator (ie, the P-mJOA MDLE subscore), which was concordant. Taken together, these data provide “very good” quality evidence for the overall validity of the PerfOMs in the assessment of DCM.

## Discussion

### Principal Findings

Smartphone apps are increasingly being used to administer clinical outcome measures in medicine. This study used consensus-based standards to assess the validity of an app designed by neurosurgeons and computer scientists from the University of Cambridge. A total of 2 lines of evidence were produced: first, a panel of correlations between the app’s tasks and established clinical comparators, and second, a panel of ratings made in accordance with prespecified hypotheses. The former produced modular evidence of construct validity and the latter a means for its overall appraisal. This type of evidence corresponds to clinical validation under the V3 framework for biometric monitoring technologies and succeeds in laboratory-based verification and analytical validation [21].

Construct validity uses comparison to other measures to assess validity. Where comparators take different approaches or contain their own limitations, validity should not be defined by traditional correlation thresholds [37]. This is applicable to DCM, where we are trying to improve disease measurement. For example, we recognize the mJOA score as a gold standard measure of disease severity, but it measures multiple constructs with limited discrimination, particularly of milder diseases. If a new measure has a correlation of 1.0 with an existing measure, it indicates that the 2 instruments are equivalent, which suggests it is unlikely to offer any improvement. For assessing construct validity, it is therefore preferable to explore expected relationships through hypothesis testing. As expected, the direction and magnitude of MoveMed correlations were most convergent between tasks and questionnaires measuring similar constructs than tasks and questionnaires measuring dissimilar constructs (eg, fast tap test vs P-mJOA > fast tap test vs WHOQOL-Bref). This is because neuromuscular tasks should correlate more with neuromuscular constructs than with non-neuromuscular ones (eg, finger dexterity vs upper extremity neuromuscular function > finger dexterity vs quality of life) and because unidimensional tasks should correlate more with other unidimensional measures than with multidimensional ones (eg, unidimensional vs unidimensional>unidimensional vs multidimensional).

To enable performance across correlations to be judged, a proportion of overall hypothesis agreement may be used [34]. After rating, 74% (67/90) and 47% (8/17) of unidimensional and multidimensional results, respectively, were deemed sufficient for construct validity in the DCM subgroup. In the absence of risk of bias factors, these data provide “very good” quality evidence for the validity of MoveMed tasks in DCM.

The standards adopted by this study have been previously used in the assessment of PerfOMs by authors of the COSMIN guidance [33]. While not originally designed for this purpose, these standards are considered to be a cornerstone in clinimetric validation and, importantly, overlap with industry guidance from the US Food and Drug Administration [17,38]. This study thus made a point to conduct and report the COSMIN Risk of Bias assessment to aid the reader in their interpretation of the rating panels (Table 2).

While construct validity testing (often criterion validity) is more commonly used by investigators, correlation coefficients require interpretation, as outlined. For similar reasons, the relative performance of instruments should not be judged solely based on the magnitude of correlation coefficients. This is reflected in clinimetric standards which instead recognize content validity as the most important arbitrator of validity and wider performance. Content validity uses stakeholder judgment and feedback to determine validity and will be further reported separately for MoveMed, following study completion. When developing and reviewing measurement instruments, understanding clinimetrics is therefore critical.

In this cohort, the impact that DCM would be expected to have on the P-mJOA and WHOQOL-Bref was similarly seen on the fast tap, hold, typing, and stand and walk MoveMed Tests. Correlations with total scores and limb-specific subscores were recorded, in accordance with prespecified expectations. The most interesting finding was the strong correlation between the P-mJOA MDUE subscore and the hold test Stability Score. This is because upper limb stability is not classically thought to be a marker of DCM. The authors attribute this finding to the composite nature of the upper limb stability construct, which includes elements of arm strength, muscle fatigue, and balance. Further studies will follow-up with more data on the subject (eg, content validity). This may very well be an example of a subclinical phenomenon that the human eye cannot catch but that mobile sensors can.

An important strength of this study is its design by individuals with formal training in clinimetrics. This is reflected in the absence of risk-of-bias factors from the COSMIN checklist in Table 2 and the study’s reporting. There is, unfortunately, a general paucity of well-designed clinimetric studies in the literature [33,34,39-41]. The use of the COSMIN manual is thus strongly encouraged by the authors. Another strength of this study was the use of PerfOMs that can collect several measurements quickly, ecologically, and longitudinally. This means that the construct should be captured more precisely, more reflective of pathology in the patient’s natural environment, and potentially more responsive to intervention. In the future, these hypotheses will be formally assessed via further clinimetric studies.

Despite its conscientious design, this study has limitations. First, standards for patient-reported methods were adapted to assess performance-based methods. This was done to overcome the absence of standardized criteria in this field and because there is precedent for it in Terwee et al [34] and the COSMIN manual [33]. Second, this study reports on 27 individuals (7 months of recruitment). The COSMIN standards are known for being rigorous (or stringent) and, ideally, at least 50 participants should be included to earn a modified Grading Of Recommendations, Assessment, Development, and Evaluations (GRADE) score of “high” [33,34,39-41]. Third, we assumed that the constructs of all WHOQOL-Bref domains would be dissimilar to the PerfOMs but this may not be the case. The WHOQOL-Bref, ultimately, contains questions on physical activity, and the relatedness of this construct to the fast tap tests and the typing tests may have been observed in Table 2. Fourth, people with a severe form of the disease may have been excluded from enrollment. This would be due to the exclusion of individuals who were unable to stand and walk without the assistance of another person. The potential risks of remote participation in this subset of individuals, however, were deemed to outweigh the benefits by the ethical committee. Further in-person research could address this limitation in the future.

### Conclusions

This study provides initial evidence for the validity of the MoveMed PerfOMs in the context of adults with DCM in the community.

## Appendix

Multimedia Appendix 1 COSMIN (Consensus-Based Standards for the Selection of Health Measurement Instruments) risk-of-bias checklist.

## Abbreviations

COSMIN: Consensus-Based Standards for the Selection of Health Measurement Instruments
DALY: disability-adjusted life year
DCM: degenerative cervical myelopathy
GRADE: Grading of Recommendations, Assessment, Development, and Evaluations
mJOA: modified Japanese Orthopaedic Association
MDLE: motor function of the lower extremities
MDUE: motor function of the upper extremities
PerfO: performance-based outcome
PerfOM: performance-based outcome measure
PRO: patient-reported outcome
PROM: patient-reported outcome measure
P-mJOA: patient-derived modified Japanese Orthopaedic Association
WHOQOL-Bref: World Health Organization Quality of Life Brief Version

## Data Availability

All data generated or analyzed during this study are included in this published paper.
