# Supplementary material for: Validity of a Smartphone App to Objectively Monitor Performance Outcomes in Degenerative Cervical Myelopathy: Preliminary Findings From a Longitudinal Observational Study
Source: JMIR Neurotechnol. 2024 Sep 9;3:e52832. doi: 10.2196/52832 (PMC12671324; doi:10.2196/52832)
Supplement: Multimedia Appendix 1 [file neuro_v3i1e52832_app1.docx]

| ***9. Hypotheses testing for construct validity*** | |  |  |  |
| --- | --- | --- | --- | --- |
| **9a. Comparison with other outcome measurement instruments (convergent validity)** | | **Rating** | **Meaning** | **Evidence** |
| 1 | Is it clear what the comparator instrument(s) measure(s)? | **Very good** | Constructs measured by the comparator instrument(s) is clear | Methods > Patient-reported comparators |
| 2 | Were the measurement properties of the comparator instrument(s) adequate? | **Very good** | Sufficient  measurement  properties of the  comparator  instrument(s) in a  population  similar to the  study population | Methods > Patient-reported comparators |
| 3 | Was the statistical method appropriate for the hypotheses to be tested? | **Very good** | Statistical  methods applied  appropriate | Methods > Statistical analysis |
|  | **Overall rating** | **Very good** |  |  |
